# The impact of accessibility to non-calcium-based phosphate binders and calcimimetics on mineral outcomes in patients receiving maintenance hemodialysis: A 10-year retrospective analysis of real-world data

**DOI:** 10.1371/journal.pone.0304649

**Published:** 2024-05-31

**Authors:** Sinee Disthabanchong, Panhathai Kasempin, Praopilad Srisuwarn, Payupol Chansomboon, Nuchcha Buachum

**Affiliations:** Faculty of Medicine, Department of Medicine, Division of Nephrology, Ramathibodi Hospital, Mahidol University, Bangkok, Thailand; RWTH Aachen University Medical Faculty: Rheinisch-Westfalische Technische Hochschule Aachen Medizinische Fakultat, GERMANY

## Abstract

**Introduction:**

Hyperphosphatemia and hyperparathyroidism are common in end-stage kidney disease and are associated with poor outcomes. In addition to adequate dialysis, medications are usually required for optimum control of serum phosphate and parathyroid hormone (PTH) levels. The use of calcium-based phosphate binders (CBPBs) and active vitamin D is associated with an increase in serum calcium and worsening vascular calcification. To overcome these limitations, non-calcium-based phosphate binders (NCBPBs) and calcimimetics have been developed. However, the coverage for these new medications remains limited in several parts of the world due to the lack of patient-level outcome data and cost. The present study examined the differences in mineral outcomes between two main categories of healthcare programs that provided different coverage for medications used to control mineral and bone disorders (MBD). The Social Security/Universal Coverage (SS/UC) program covered only CBPBs and active vitamin D, whereas the Civil Servant/State Enterprise (CS/SE) program provided coverage of CBPBs, active vitamin D, NCBPBs, and calcimimetics.

**Methods:**

This 10-year retrospective cohort study examined the differences in mineral outcomes between two healthcare programs in maintenance hemodialysis patients. The differences in serum calcium, phosphate, and PTH levels, as well as the aortic arch calcification score, were analyzed according to dialysis vintage by linear mixed-effects regression analyses. The difference in the composite outcome of severe hyperparathyroidism and parathyroidectomy was analyzed by the Cox-proportional hazard regression model.

**Results:**

714 patients were included in the analyses (full cohort). Of these patients, 563 required at least one type of medication to control MBD (MBD medication subgroup). Serum calcium, phosphate, and the proportions of patients with hypercalcemia and hyperphosphatemia were substantially higher in the SS/UC group compared with the CS/SE group after appropriate adjustments for confounders in both the full cohort and the MBD medication subgroup. These findings were confirmed in propensity-score matched analyses. Higher parathyroid hormone levels and a higher rate of the composite endpoint of severe hyperparathyroidism and parathyroidectomy were also observed in the SS/UC group. A more rapid progression of aortic arch calcification was suggested in the SS/UC group, but between-group changes were not significant.

**Conclusion:**

Patients under the healthcare program that did not cover the use of NCBPBs and calcimimetics showed higher serum calcium and phosphate levels and a more rapid progression of hyperparathyroidism. The difference in the progression of vascular calcification could not be confirmed in the present study.

## Introduction

Mineral and bone disorder (MBD) in chronic kidney disease (CKD) comprises abnormalities in mineral metabolites and hormones, including serum calcium, phosphate, fibroblast growth factor-23, and parathyroid hormone (PTH), as well as bone disorder and vascular calcification [1]. In end-stage kidney disease (ESKD), almost all patients develop metabolic derangements including phosphate retention and hyperparathyroidism. The use of phosphate binders, active vitamin D, and calcimimetics is usually required for optimal control of serum phosphate and PTH.

Calcium-based phosphate binders (CBPBs) were the most commonly used phosphate binders in the 1980s and 1990s [2]. However, the recognition and awareness of a high prevalence of vascular calcification and cardiovascular disease (CVD) among ESKD patients have prompted investigations into the underlying mechanisms [3,4]. In vitro, studies have confirmed the roles of increased extracellular calcium and phosphate in the development and progression of vascular calcification [5]. Because the use of CBPBs is associated with a more rapid progression of vascular calcification, therefore, non-calcium-based phosphate binders (NCBPBs) are the preferred phosphate-binding agents in several upper-middle-income and most high-income countries [6]. However, due to the lack of strong evidence regarding patient outcomes beyond the reduction in serum calcium and slowing the progression of vascular calcification, several national healthcare programs impose restrictions on the use of NCBPBs [7,8].

Active vitamin D has been used to lower PTH levels, but at the expense of an increase in serum calcium through enhanced gastrointestinal calcium absorption, especially when used in combination with CBPBs [9,10]. Calcimimetics have been developed to overcome this limitation. Calcimimetics lowers the threshold for calcium-sensing receptor activation by extracellular calcium in the parathyroid glands, resulting in a reduction in PTH release as well as serum calcium [11]. Since the approval of the first-generation calcimimetics, cinacalcet, a decline in the rate of parathyroidectomy has been reported in several developed regions of the world [12]. The attenuation of the progression of vascular calcification has also been demonstrated [13]. Similar to NCBPBs, the favorable impact of calcimimetics on patient outcomes beyond the reduction in the rate of parathyroidectomy and vascular calcification has not been confirmed [14]. Due to this reason and the cost of the drug, the coverage and availability of calcimimetics remain limited.

Thailand has two main categories of healthcare programs that provide different coverage for medications used to control MBD. The Social Security and Universal Coverage (SS/UC) programs provide coverage only for the medications listed in the National List of Essential Drugs, which does not include NCBPBs and calcimimetics. To use these drugs, patients are required to make the full payment for the cost. The Civil Servant and State Enterprise (CS/SE) programs cover the use of NCBPBs and calcimimetics if they are deemed necessary by the primary nephrologist. Ramathibodi Hospital is a large tertiary/quaternary care public university hospital that serves patients under both categories of healthcare programs, providing a unique opportunity for investigating the differences in mineral outcomes. This retrospective cohort study examined the differences in serum calcium, phosphate, the rate of parathyroidectomy, and the progression of vascular calcification in patients receiving maintenance hemodialysis (HD) between the two groups of national healthcare programs.

## Materials and methods

### Ethics approval

This retrospective observational study was approved by the Human Research Ethics Committee of the Faculty of Medicine, Ramathibodi Hospital, Mahidol University, Bangkok, Thailand (Approval number: MURA2022/356) and was conducted according to the Declaration of Helsinki. The study has been granted an exemption from requiring written informed consent. The patients’ data were accessed through the hospital’s electronic medical record system between July 2022 and September 2023. During this data collection period, the authors had access to the information that could identify individual patients. The data were analyzed anonymously.

### Participants

**Fig 1:**
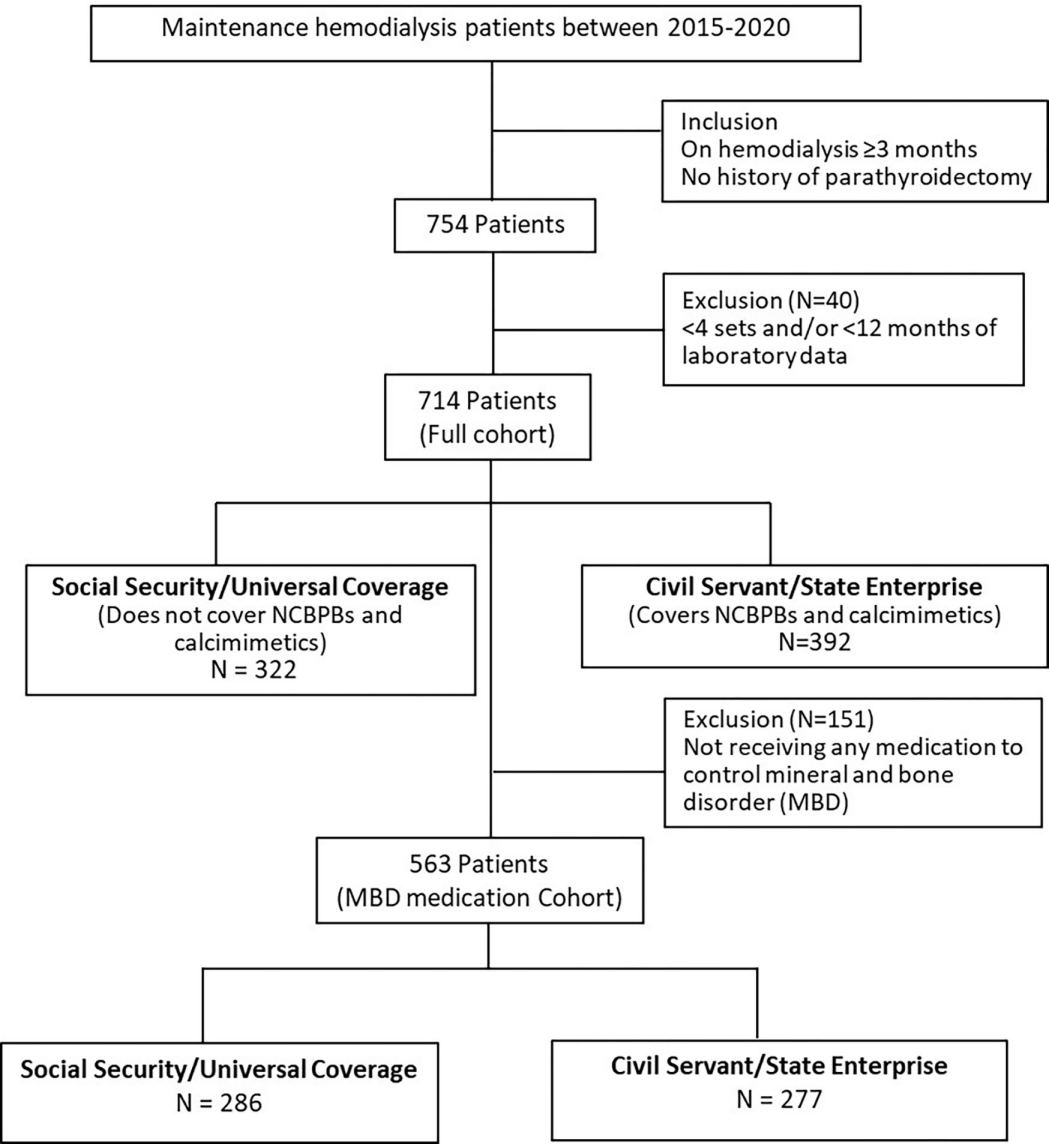
Study flow chart.

The details of patient selection are shown in Fig 1. All patients receiving maintenance HD at Ramathibodi Hospital between 2015 and 2020 were identified. The inclusion criteria were: (1) age ≥ 18 years; (2) having been on HD for ≥ 3 months. Patients who had undergone parathyroidectomy were excluded. Seven hundred fifty-four patients met the eligibility criteria. Forty patients had fewer than 4 sets or less than 12 months of available laboratory data and were excluded. Seven hundred fourteen patients were included in the final analysis. All patients received 4-hour sessions, twice or thrice weekly HD or hemodiafiltration using a high-flux polysulfone dialyzer with a surface area between 1.8–2.1 m^2^. The dialysate sodium concentration ranged between 136–140 mEq/L, potassium 2–3 mEq/L, calcium 2.5–3.5 mEq/L, magnesium 0.7 mEq/L, and bicarbonate 32–36 mEq/L. The standard of care for all HD patients followed the 2014 and 2022 Thailand Hemodialysis Clinical Practice Guideline recommendations [15].

### Healthcare programs and coverage of MBD medication

The population under the SS/UC program is younger with fewer comorbidities because the SS system started in Thailand in 1990 and covers individuals who are employees of Thai-registered companies until the age of 60. The UC system was established later in 2002 and covers Thais who are not eligible for any other healthcare programs. On the other hand, the CS/SE program has been in existence for more than 10 decades, with coverage extending to biological parents. The coverage is lifelong for CS and until the age of 60 for SE. This results in the population under the CS/SE program being significantly older with more substantial comorbidities.

The MBD medications available during the study period were calcium carbonate, calcium acetate, calcitriol, alfacalcidol, sevelamer carbonate, lanthanum carbonate, and cinacalcet. Both categories of health programs covered the use of CBPBs, calcitriol, and alfacalcidol, whereas only the CS/SE program covered the use of NCBPBs and calcimimetics. The coverage for HD, other medications including erythropoietin and intravenous iron, and parathyroidectomy were comparable between both programs.

Approximately 5% of the patients in the SS/UC group received NCBPBs or calcimimetics because they were willing to pay for the medications. Since not all patients required the use of medications to control serum phosphate and PTH, the subgroup of patients that received at least one type of medication was also analyzed. To qualify for receiving MBD medication, the medicine had to be prescribed for at least 2 consecutive months. To ensure that calcium was prescribed for phosphate binding, the amount of elemental calcium had to be at least 1000 mg/day, and the medication was prescribed at least twice daily after meals. For active vitamin D, the dose of calcitriol had to be at least 0.75 μg/week, and alfacalcidol 1.5 μg/week [16].

### Follow-up and outcomes

All patients were followed until death, loss to follow-up, kidney transplantation, parathyroidectomy, or the end of 2022. The primary outcomes were the differences in serum calcium, phosphate, PTH, and the composite outcome of parathyroidectomy and severe hyperparathyroidism between the two groups of healthcare programs. The outcome of severe hyperparathyroidism required the presence of a progressive increase in PTH levels with at least two PTH values ≥1500 pg/mL within 12 months. The secondary outcome was the difference in the progression of aortic arch calcification (AAC). Due to substantial differences in baseline demographic data between the two groups, propensity-score matchings were performed before the determination and analyses of AAC. The matchings were performed in the full cohort and in two subgroups: patients who received any type of MBD medication and patients who received NCBPBs or calcimimetics. The latter subgroup involved the subgroup of patients in the CS/SE program who received NCBPBs or calcimimetics and the subgroup of patients in the SS/UC program who never received NCBPBs or calcimimetics.

### Biochemical data

All outpatient predialysis laboratory data were collected, excluding the data during the last 6–12 months before death. Serum calcium was corrected based on the following equation: corrected calcium (mg/dl) = serum calcium (mg/dl) + [(40 –serum albumin (g/l))/10 × 0.8]. Baseline laboratory data in Table 1 represented the first 12-month average values after enrollment. For changes in mineral parameters over time, laboratory data for each patient were averaged according to the interval dialysis vintage. Hypercalcemia, hyperphosphatemia, and hyperparathyroidism were determined for each laboratory value, and the proportion (percentage) of abnormal laboratory values was calculated for each interval of dialysis vintage.

**Table 1:** Baseline characteristics according to two categories of healthcare programs.

Parameters	All patients (N = 714)			Subgroup of patients who received MBD Medication (N = 563)		
	SS/UC N = 322	CS/SE N = 392	*P*	SS/UC N = 286	CS/SE N = 277	*P*
Age (year)	42.42±12.3	67.63±13.23	<0.001	42±12.32	64.65±13.55	<0.001
Male sex (n/%)	173 (53.7)	174 (44.4)	0.01	150 (52.4)	118 (42.6)	0.02
Body mass index (kg/m^2^)	23.43±4.76	24.41±6.19	0.02	23.42±4.76	24.72±6.35	0.006
Diabetes mellitus (n/%)	55 (17.1)	202 (51.5)	<0.001	46 (16.1)	129 (46.6)	<0.001
CVD (n/%)	48 (14.9)	148 (37.8)	<0.001	40 (14)	90 (32.5)	<0.001
Hypertension (n/%)	284 (88.2)	354 (90.3)	0.36	252 (88.1)	248 (89.5)	0.59
Dyslipidemia (n/%)	81 (25.1)	179 (45.7)	<0.001	66 (23.1)	116 (41.9)	<0.001
Dialysis data						
Twice weekly HD (n/%)	74 (23)	79 (20.2)	0.27	64 (22.3)	56 (20.2)	0.64
Hemodiafiltration (n/%)	10 (3.1)	14 (3.6)	0.73	8 (2.8)	12 (4.3)	0.33
Kt/V	1.82±0.33	1.89±0.36	0.12	1.82±0.37	1.88±0.39	0.19
nPCR	1.29±0.38	1.23±0.26	0.08	1.28±0.27	1.22±0.26	0.09
Arteriovenous access (n/%)			<0.001			<0.001
Arteriovenous fistula	242 (75.2)	142 (36.2)		218 (76.2)	105 (37.9)	
Arteriovenous graft	42 (13)	141 (36)		35 (12.2)	101 (36.5)	
Catheter	38 (11.8)	109 (27.9)		33 (11.5)	71 (25.6)	
MBD Medications (n/%)						
CBPBs	243 (75.5)	154 (39.3)	<0.001	243 (85)	154 (55.6)	<0.001
NCBPBs	12 (3.7)	162 (41.3)	<0.001	12 (4.2)	162 (58.5)	<0.001
Active vitamin D	210 (65.2)	137 (34.9)	<0.001	210 (73.4)	137 (49.5)	<0.001
Calcimimetics	8 (2.5)	100 (25.5)	<0.001	8 (2.8)	100 (36.1)	<0.001
NCBPBs or calcimimetics	17 (5.3)	191 (48.7)	<0.001	17 (5.9)	191 (69)	<0.001
Any MBD medications	286 (88.8)	277 (70.7)	<0.001	286 (100)	277 (100)	-
Laboratory data						
Hemoglobin (g/dL)	10.68±1.66	10.93±1.29	0.02	10.67±1.67	10.97±1.34	0.02
Albumin (g/L)	37.43±3.6	34.08±3.97	<0.001	37.48±3.62	34.57±3.79	<0.001
Calcium (mg/dL)	9.87±0.74	9.76±0.66	0.04	9.91±0.73	9.77±0.69	0.02
Phosphate (mg/dL)	5.45±1.38	4.37±1.29	<0.001	5.52±1.38	4.62±1.29	<0.001
Alkaline phosphatase (u/L)	80.3 (61.7–114)	86.7 (68.3–116)	0.03	80.7 (62.5–115)	91.9 (69.3–119)	0.04
PTH (pg/mL)	466 (248–895)	229 (109–447)	<0.001	504 (265–980)	309 (148–548)	<0.001
Creatinine (mg/dL)	10.66±2.99	7.64±2.9	<0.001	10.81±3	8.29±2.9	<0.001

Laboratory data were 12-month average values. SS/UC, Social Security/Universal Coverage; CS/SE, Civil Servant/State Enterprise; MBD, mineral and bone disorder; CVD, cardiovascular disease; HD, hemodialysis; CBPBs, calcium-based phosphate binders; NCBPBs, non-calcium-based phosphate binders; PTH, parathyroid hormone.

### Aortic arch calcification

AAC was determined on the posteroanterior chest X-ray using the validated semiquantitative method devised by Ogawa et al, chosen for its appropriateness and practicality in this situation. A high correlation with the calcification score obtained from computed tomography has been described [17]. The Ogawa scoring system has also been successfully used to assess the progression of AAC over time [18,19]. Briefly, the AAC score was determined using a circular scale with 16 circumferences that divided the aortic arch into 16 sections. Each section with calcification was counted, and the counts were then combined, yielding a final score ranging between 0–16. All available posteroanterior chest X-rays were reviewed, and those with adequate quality were considered. If multiple X-rays of adequate quality were available during the same period, the AAC scores were averaged. Two experts independently reviewed each X-ray, and they were blinded to clinical data. The correlation between the two experts was > 0.9.

### Statistical analysis

Differences between the two groups were compared using Student’s t-test, Mann-Whitney U test, or Chi-square test. To determine the longitudinal changes in mineral parameters within the same group or between two groups, linear mixed models that allowed both fixed and random effects were performed with appropriate adjustments for unbalanced baseline factors. The differences in the composite outcome of parathyroidectomy and severe hyperparathyroidism were determined by a multivariate Cox-proportional Hazard Regression model before and after inverse probability weighting (IPW) with trimming at the 70^th^ percentile. Propensity- score matchings were performed according to age, sex, and diabetes with a matching tolerance of 0.07 (without replacement and with maximized performance). A p-value <0.05 was considered statistically significant. All statistical analyses were performed using IBM SPSS Statistics for Windows, version 26 (IBM Corp., Armonk, N.Y., USA).

## Results

### Baseline characteristics

The baseline characteristics of all patients and the subgroup of patients who received MBD medication are shown in Table 1. The population under the SS/UC program was substantially younger and mostly male. Therefore, they had fewer comorbidities with higher hemoglobin, serum albumin, calcium, phosphate, and PTH. The arteriovenous access was mostly an arteriovenous fistula rather than the catheter. The differences in baseline data were due to the reasons mentioned in the Materials and Methods section (Healthcare programs and coverage of MBD medication).

To identify appropriate covariates for adjustments in the comparisons of outcomes between the two healthcare programs, the relationships between baseline demographic data, including age, sex, and diabetes, with other demographic and laboratory data were explored (S1–S4 Tables). Age was negatively associated with serum albumin, calcium, phosphate, PTH, and creatinine and positively associated with diabetes, CVD, and dyslipidemia. Male participants exhibited substantially higher serum albumin, phosphate, and creatinine but lower serum calcium. Patients with diabetes were older and more likely to have CVD, dyslipidemia, and catheter as HD access. They also had higher body mass index and hemoglobin, but lower serum albumin, calcium, phosphate, PTH, and creatinine.

Since age and sex were highly correlated with other demographic and laboratory data, they were chosen as covariates for adjustment in model 1 of the mixed model analyses. Although aging and diabetes were highly interacted, diabetes could exert an independent effect on the mineral outcomes, especially vascular calcification, therefore, it was chosen as the third covariate in model 2. Propensity-score matchings were also performed according to age, sex, and diabetes.

### Mineral outcomes of the full cohort

**Fig 2:**
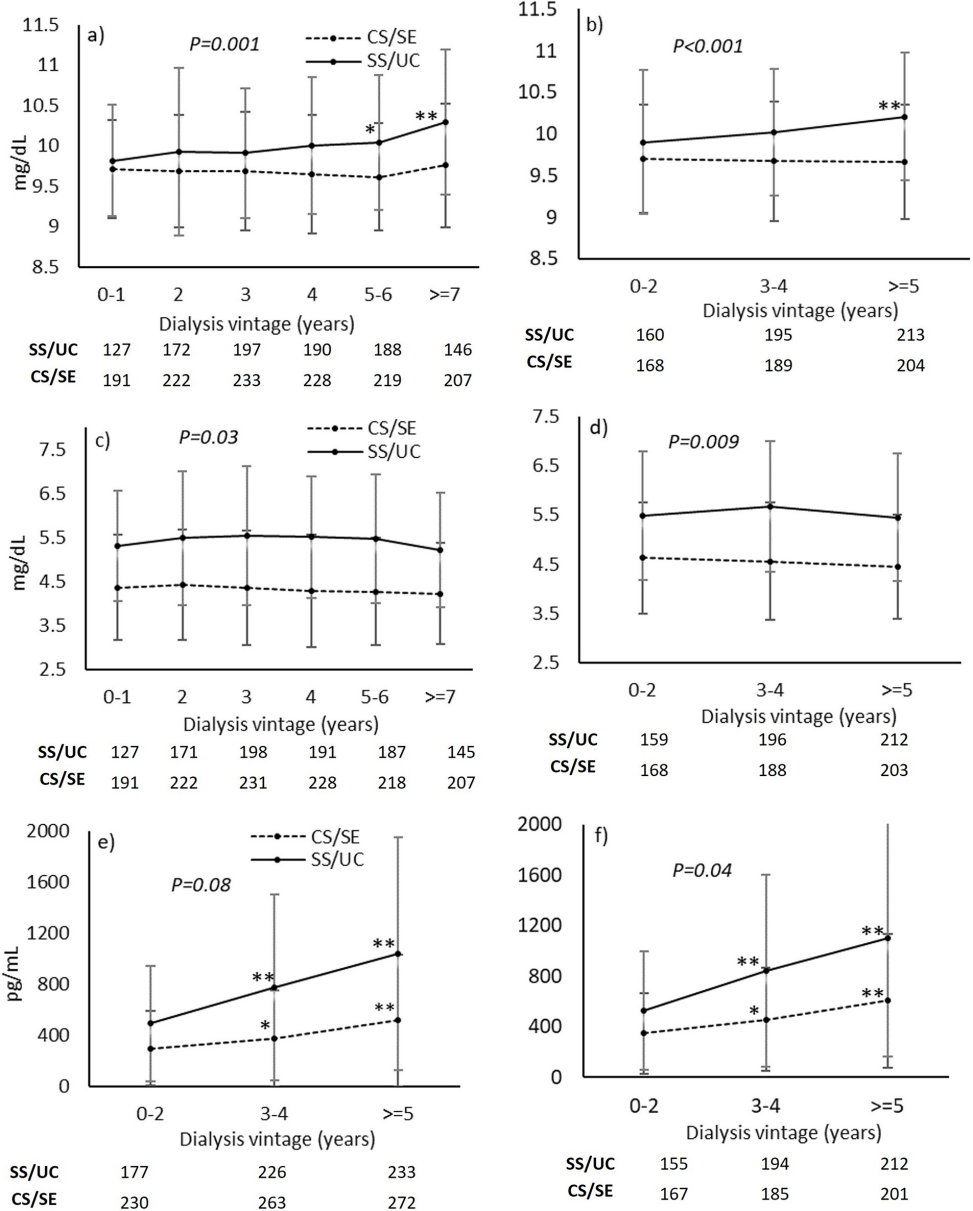
Mineral parameters according to dialysis vintage. a) Serum calcium; c) serum phosphate and e) parathyroid hormone for all patients. b) serum calcium; d) serum phosphate and f) parathyroid hormone for the subgroup of patients who received MBD medication. The number of available laboratory data is shown below each graph. CS/SE, Civil Servant/State Enterprise; SS/UC, Social Security/Universal Coverage; MBD, mineral and bone disorder. *P<0.05 and **P<0.001 vs. Year 0–1 or Year 0–2 of the same group. P-values in the graph represent the significance of between-group changes after adjustment for age, sex, and diabetes (Model 2).

Table 2 shows the changes in mineral parameters for all patients. Serum calcium was significantly and invariably higher in the SS/UC group in all models. Similarly, serum phosphate was also significantly higher in the SS/UC group in all models, although the significance was attenuated in the adjusted models. Between-group changes in PTH levels were significant in the unadjusted model and model 1, but the significance was lost in model 2. Within-group comparisons revealed a substantial increase in serum calcium in the SS/UC group at 5–6 years and ≥7 years of dialysis vintage compared with 0–1 years, whereas stable serum calcium was observed in the CS/SE group throughout the observational period (Fig 2A). Serum phosphate was stable throughout the study period in both groups (Fig 2C). On the other hand, PTH levels increased progressively over time in both groups (Fig 2E).

**Table 2:** Linear mixed models of mineral outcomes according to dialysis vintage for all patients.

Labs	Program		Dialysis vintage (years)						Between-group Comparisons (SS/UC vs. CS/SE) Mean Difference (95% Confidence Interval)					
			0–1	2	3	4	5–6	≥ 7	Unadjusted	P	Model 1	P	Model 2	P
Calcium (mg/dL)	-	SS/UC	9.82 (9.67,9.97)	9.93 (9.8,10.1)	9.91 (9.79,10)	10 (9.87,10.1)	10.04 (9.9,10.2)	10.29 (10.2,10.4)	0.31 (0.22,0.41)	0.001	0.3 (0.16,0.43)	0.001	0.26 (0.13,0.4)	0.001
		CS/SE	9.71 (9.61,9.81)	9.69 (9.6,9.78)	9.68 (9.59,9.77)	9.65 (9.56,9.74)	9.61 (9.52,9.71)	9.76 (9.66,9.86)						
Phosphate (mg/dL)	-	SS/UC	5.31 (5.06,5.56)	5.49 (5.27,5.7)	5.54 (5.34,5.74)	5.52 (5.31,5.72)	5.47 (5.27,5.68)	5.22 (4.99,5.46)	1.13 (0.97,1.3)	0.001	0.23 (0.02,0.44)	0.033	0.24 (0.03,0.46)	0.028
		CS/SE	4.36 (4.19,4.54)	4.43 (4.27,4.59)	4.36 (4.2,4.52)	4.29 (4.13,4.45)	4.28 (4.11,4.44)	4.23 (4.06,4.44)						
Labs	Program		0–2		3–4		≥ 5		Unadjusted	P	Model 1	P	Model 2	P
PTH (pg/mL)	-	SS/UC	494 (384,603)		779 (682,876)		1041 (945,1136)		440 (351,529)	0.001	130 (10.4,250)	0.033	108 (-13.3,229)	0.08
		CS/SE	297 (244,350)		377 (327,426)		516 (467,565)							
Hypercalcemia (%)	> 10.5 mg/dL	SS/UC	16.9 (11.9,21.8)		22.3 (17.9,26.8)		33.8 (29.4,38.2)		11.43 (7.58,15.3)	0.001	8.34 (2.98,13.7)	0.002	7.13 (1.73,12.5)	0.01
		CS/SE	12.9 (9.73,16.1)		13.3 (10.3,16.2)		15.3 (12.4, 18.2)							
	> 11 mg/dL	SS/UC	8.67 (4.89,12.4)		10.7 (7.27,14.1)		18.8 (15.4,22.1)		7.98 (5.43,10.5)	0.001	7.74 (4.18,11.3)	0.001	7.01 (3.42,10.6)	0.001
		CS/SE	4.08 (2.14,6.01)		4.87 (3.07,6.68)		6.9 (5.15,8.7)							
Hyperphosphatemia (%)	> 4.5 mg/dL	SS/UC	66.4 (61.3,71.4)		69.4 (64.9,74)		67.1 (62.6,71.6)		28 (23.6,32.4)	0.001	6.59 (0.8,12.4)	0.026	6.84 (0.96,12.7)	0.023
		CS/SE	41.1 (36.6,45.5)		40.3 (36.2,44.5)		39 (34.9.43.1)							
	> 5 mg/dL	SS/UC	53.6 (48.1,59.1)		58.1 (53.1,63)		55.6 (50.7,60.5)		28.31 (23.9,32.7)	0.001	6.51 (0.77,12.3)	0.026	6.66 (0.83,12.5)	0.025
		CS/SE	28.5 (24.5, 32.5)		27.9 (24.1, 31.6)		26.7 (23,30.3)							
Hyperparathyroidism (%)	> 600 pg/mL	SS/UC	25.84 (20.61,31.08)		43.83 (38.98,48.68)		54.79 (49.93,59.65)		21.79 (17.46,26.1)	0.001	6.39 (0.58,12.2)	0.031	5.49 (-0.37,11.4)	0.066
		CS/SE	12.6 (8.97,16.23)		19.76 (16.32,23.2)		29.43 (26,32.87)							
	>1000 pg/mL	SS/UC	11.6 (6.91,16.29)		23.8 (19.46,28.14)		36.56 (32.21,40.91)		16.1 (12.6,19.7)	0.001	5.82 (0.99, 10.6)	0.017	4.88 (0.02,9.74)	0.049
		CS/SE	3.98 (1.39,6.56)		7.42 (4.97,9.88)		15.3 (12.85,17.75)							

Data are presented as mean (95% confidence interval); Model 1, adjusted for age and sex; Model 2, adjusted for age, sex, and diabetes mellitus; SS/UC, Social Security/Universal Coverage; CS/SE, Civil Servant/State Enterprise; PTH, parathyroid hormone; MBD, mineral and bone disorder.

**Fig 3:**
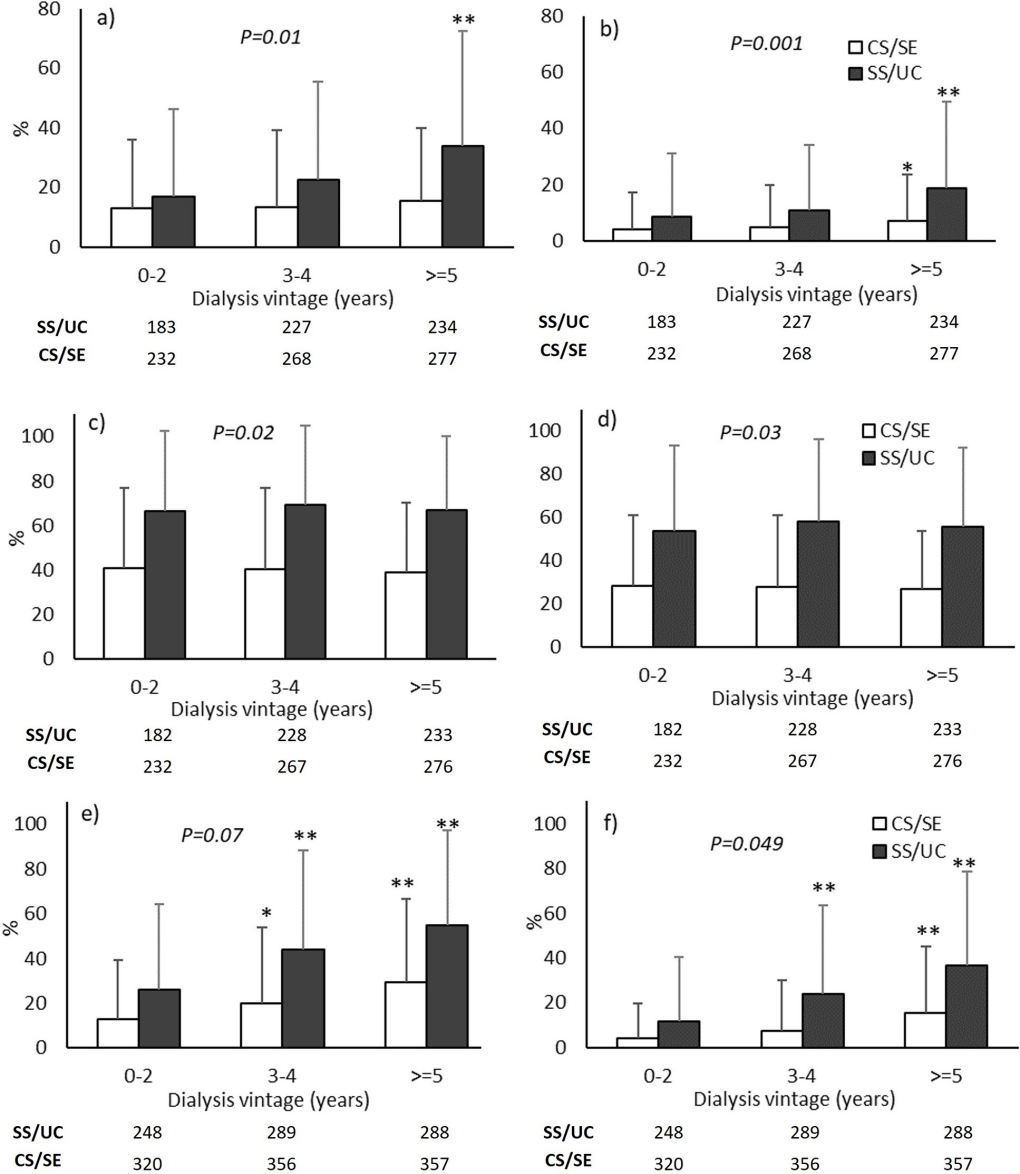
Proportions of patients with abnormal mineral parameters of the full cohort. a) serum calcium >10.5 mg/dL; b) serum calcium >11 mg/dL; c) serum phosphate >4.5 mg/dL; d) serum phosphate >5 mg/dL; e) parathyroid hormone levels >600 pg/mL; f) parathyroid hormone levels >1000 pg/mL. The number of available laboratory data is shown below each graph. SS/UC, Social Security/Universal Coverage; CS/SE, Civil Servant/State Enterprise. *P<0.05 and **P<0.001 vs. Year 0–2 of the same group. P-values in the graph represent the significance of between-group changes after adjustment for age, sex, and diabetes (Model 2).

The proportions of patients with hypercalcemia (serum calcium >10.5 mg/dL and >11 mg/dL) and hyperphosphatemia (serum phosphate >4.5 mg/dL and >5 mg/dL) were significantly higher in the SS/UC group in all models (Table 2). The proportion of patients with PTH levels >600 pg/mL was significantly higher in the SS/UC group in the unadjusted model and model 1, but not in model 2. Nevertheless, the proportion of patients with PTH >1000 pg/mL was significantly higher in the SS/UC group in all models. Within-group comparisons revealed a significant increase in the proportion of patients with hypercalcemia at ≥5 years compared with 0–2 years of dialysis vintage in the SS/UC group. In the CS/SE group, the proportion of patients with serum calcium >10.5 mg/dL was stable throughout the observational period, but a substantial increase was observed with serum calcium >11 mg/dL (Fig 3A and 3B). The proportions of patients with hyperphosphatemia were stable throughout the observational period in both groups (Fig 3C and 3D). A significant increase in the proportions of patients with PTH >600 pg/mL and >1000 pg/mL with increasing dialysis vintage was observed in both groups (Fig 3E and 3F).

### Mineral outcomes of the subgroup of patients who received MBD medication

Since not all patients received medications to control MBD, analyses were also performed in the subgroup of patients who received at least one MBD medication (Table 3, Figs 2B, 2D, 2F and S1). Because the number of patients was lower in the subgroup, the duration of dialysis vintage was categorized into only 3 intervals (0–2 years, 3–4 years, and ≥ 5 years). The findings in the subgroup were similar to the full cohort, with few exceptions. Between-group changes in PTH levels were substantially different in all models. However, between-group differences in the proportions of patients with PTH >600 pg/mL or >1000 pg/mL were significant only in the unadjusted models.

**Table 3:** Linear mixed models of mineral outcomes for the subgroup of patients who received MBD medication.

Labs		Program	Dialysis vintage (years)			Between-group Comparisons (SS/UC vs. CS/SE) Mean Difference (95% Confidence Interval)					
			0–2	3–4	≥ 5	Unadjusted	P	Model 1	P	Model 2	P
Calcium (mg/dL)	-	SS/UC	9.9 (9.78,10)	10 (9.91,10.1)	10.2 (10.1,10.3)	0.38 (0.27,0.49)	<0.001	0.34 (0.2,0.49)	<0.001	0.32 (0.17,0.46)	<0.001
		CS/SE	9.7 (9.6,9.81)	9.67 (9.57,9.77)	9.67 (9.57,9.76)						
Phosphate (mg/dL)	-	SS/UC	5.49 (5.28,5.69)	5.67 (5.49,5.86)	5.45 (5.28,5.63)	0.99 (0.8,1.17)	<0.001	0.3 (0.07,0.52)	0.011	0.31 (0.08,0.54)	0.009
		CS/SE	4.62 (4.45,4.8)	4.56 (4.4,4.72)	4.45 (4.29,4.6)						
PTH (pg/mL)	-	SS/UC	528 (407,649)	846 (738,954)	1099 (996,1202)	416 (311,522)	<0.001	162 (24.5,300)	0.021	148 (9.51,287)	0.036
		CS/SE	348 (282,414)	460 (397,522)	608 (548,668)						
Hypercalcemia (%)	> 10.5 mg/dL	SS/UC	18.9 (13.5,24.4)	24 (19,28.9)	35.8 (31,40.5)	12.2 (7.68,16.7)	<0.001	9.22 (3.21,15.2)	0.003	8.52 (2.46,14.6)	0.006
		CS/SE	14.2 (10.3,18.2)	14.8 (11,18.5)	16.2 (12.6,19.8)						
	> 11 mg/dL	SS/UC	9.56 (5.38,13.7)	11.7 (7.87,15.4)	19.8 (16.1,23.4)	8.63 (5.59,11.7)	<0.001	8.25 (4.19,12.3)	<0.001	7.8 (3.71,11.9)	<0.001
		CS/SE	4.6 (2.19,7.02)	5.29 (3.02,7.57)	7.23 (5.04,9.42)						
Hyperphosphatemia (%)	> 4.5 mg/dL	SS/UC	68.4 (63.2,73.6)	72.7 (67.9,77.4)	68.3 (63.7,72.8)	23.3 (18.5,28)	<0.001	6.95 (0.89,13)	0.025	7.38 (1.25,13.5)	0.018
		CS/SE	47.1 (42,52.3)	47 (42.1,51.8)	45.2 (40.5,49.9)						
	> 5 mg/dL	SS/UC	57.1 (51.2,62.9)	60.7 (55.4,66)	57 (51.9,62)	25.2 (20.3,30.2)	<0.001	6.8 (0.5,13.1)	0.034	6.96 (0.59,13.3)	0.032
		CS/SE	33.7 (28.9,38.5)	33.1 (28.6,37.7)	30.9 (26.5,35.2)						
Hyperparathyroidism (%)	> 600 pg/mL	SS/UC	30.8 (24.2,37.5)	50.6 (44.6,56.5)	59.1 (53.4,64.8)	23.4 (17.5,29.3)	<0.001	4.75 (-2.81,12.3)	0.217	4.2 (-3.44,11.8)	0.281
		CS/SE	15.7 (10.4,20.9)	25.3 (20.3,30.3)	35.4 (30.6,40.2)						
	>1000 pg/mL	SS/UC	13.7 (7.45,19.9)	28.9 (23.3,34.5)	40.1 (34.8,45.5)	19.5 (14.3,24.7)	<0.001	5.42 (-1.32,12.2)	0.115	4.54 (-2.25,11.3)	0.189
		CS/SE	5.95 (2.15,9.75)	8.81 (5.2,12.4)	17.8 (14.4,21.3)						

Data are presented as mean (95% confidence interval); Model 1, adjusted for age and sex; Model 2, adjusted for age, sex, and diabetes mellitus; SS/UC, Social Security/Universal Coverage; CS/SE, Civil Servant/State Enterprise; PTH, parathyroid hormone; MBD, mineral and bone disorder.

### The composite outcome of parathyroidectomy and severe hyperparathyroidism

**Fig 4:**
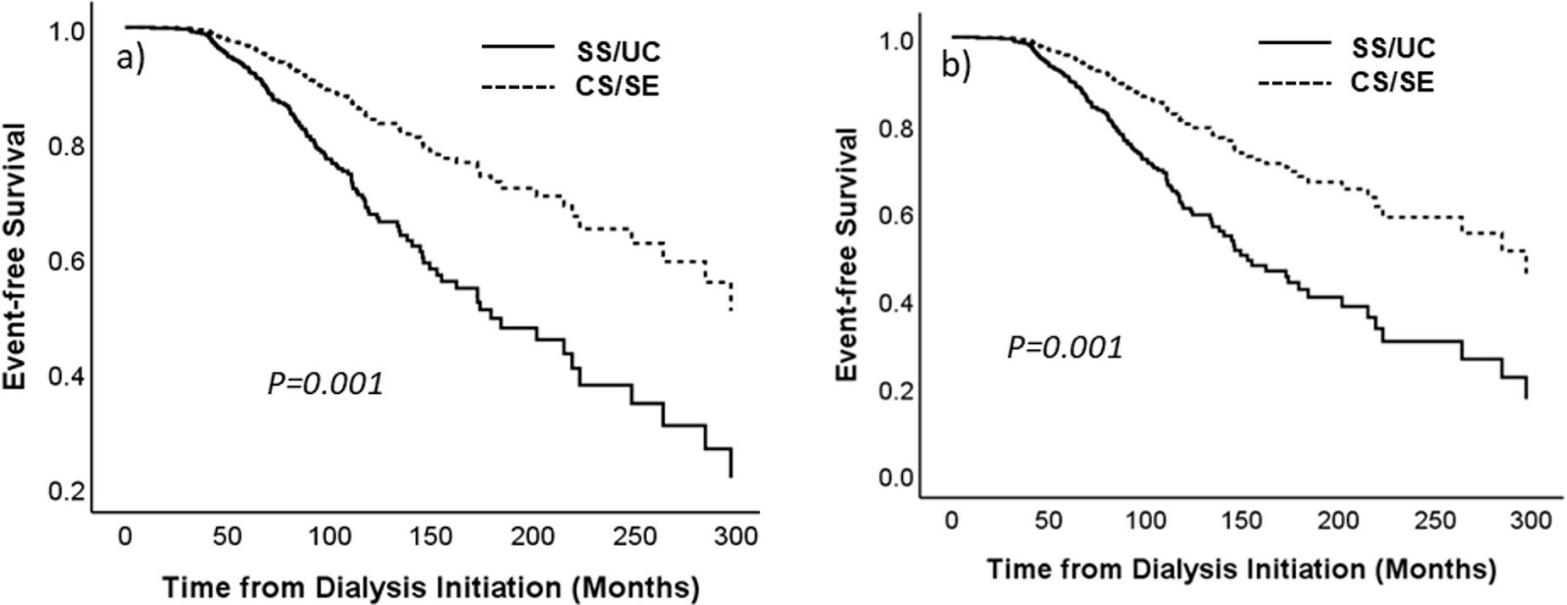
Multivariate Cox proportional hazards regression models of the composite outcome of parathyroidectomy and severe hyperparathyroidism. a) all patients; b) subgroup of patients who received MBD medication. Adjusted for age at dialysis initiation, sex, and diabetes mellitus. MBD, mineral and bone disorder; CS/SE, Civil Servant/State Enterprise; SS/UC, Social Security/Universal Coverage.

Multivariate Cox proportional hazards regression models of the composite outcome for all patients and for the subgroup of patients who received MBD medication are shown in Table 4 and Fig 4. The observational period began at the date of dialysis initiation and ended at the occurrence of an event. The average follow-up period was 84 ± 45.6 months for the SS/UC group and 88.6 ± 51.8 months for the CS/SE group (P = 0.21). In the unweighted model, younger age at dialysis initiation and SS/UC health program were independently associated with the outcome. After inverse probability weighting, younger age, not having diabetes, and SS/UC health program were independently associated with the outcome. The male sex was identified as an independent predictor of the outcome only in the weighted analysis of the full cohort.

**Table 4:** Multivariate Cox proportional hazards regression models for the composite outcome of parathyroidectomy and severe hyperparathyroidism.

Variables	All patients				Subgroup of patients who received MBD medication			
	Unweighted		After IPW		Unweighted		After IPW	
	Odds Ratio	*P*	Odds Ratio	*P*	Odds Ratio	*P*	Odds Ratio	*P*
Age at dialysis initiation	0.98 (0.96–0.99)	0.001	0.97 (0.97–0.98)	<0.001	0.98 (0.97–0.99)	0.011	0.99 (0.98–0.99)	<0.001
Sex (M vs. F)	1.22 (0.88–1.7)	0.236	1.19 (1.05–1.35)	0.006	1.2 (0.86–1.68)	0.277	1.06 (0.95–1.19)	0.265
DM (Yes vs. No)	0.78 (0.47–1.27)	0.317	0.77 (0.6–0.99)	0.041	0.78 (0.47–1.28)	0.324	0.66 (0.52–0.84)	0.001
Health Program (SS/UC vs. CS/SE)	2.26 (1.42–3.58)	0.001	3.16 (2.22–4.5)	<0.001	2.25 (1.42–3.54)	0.001	3.81 (2.72–5.34)	<0.001

Data are presented as Odds ratio (95% confidence interval); IPW, inverse probability weighting; M, male; F, female; DM, diabetes mellitus SS/UC, Social Security/Universal Coverage; CS/SE, Civil Servant/State Enterprise.

### Mineral outcomes and aortic arch calcification in propensity-score matched subgroups

AAC was analyzed in three propensity-score-matched subgroups: 232 patients from the full cohort; 204 from the subgroup of any MBD medication; and 182 from the subgroup of NCBPBs and calcimimetics. The latter subgroup involved the subgroup of patients in the CS/SE program who received NCBPBs or calcimimetics and the subgroup of patients in the SS/UC program who never received NCBPBs or calcimimetics. Baseline demographics, laboratory data, and AAC scores were largely comparable between the two groups of health programs (S5 Table). Patients under the SS/UC health program were more likely to have an arteriovenous fistula and less likely to have an arteriovenous graft compared with the CS/SE group. This difference was likely due to the requirement of co-payment for arteriovenous graft surgery before 2015 in the SS/UC healthcare program.

The changes in serum calcium, phosphate, and the proportions of patients with hypercalcemia and hyperphosphatemia are shown in Table 5. Serum calcium and the proportions of patients with hypercalcemia were significantly higher in the SS/UC group compared with the CS/SE group in all propensity-score-matched subgroups. The difference in serum phosphate was significant only in the subgroup of any MBD medication. No significant differences in the proportions of patients with hyperphosphatemia were observed in any of the subgroups.

**Table 5:** Linear mixed models of mineral outcomes for the propensity-score-matched subgroups.

Labs		Program	Dialysis vintage (years)		Within-group comparisons	*P*	Between-group Comparisons (SS/UC vs. CS/SE)	*P*
			0–3	≥4				
All patients (N = 232)								
Calcium (mg/dL)	-	SS/UC	9.87 (9.68,10.1)	10.15 (9.98,10.3)	0.28 (0.02,0.55)	0.03	0.34 (0.14,0.54)	0.001
	-	CS/SE	9.68 (9.51,9.86)	9.71 (9.55,9.86)	0.02 (-0.21,0.26)	0.84		
Phosphate (mg/dL)	-	SS/UC	5.14 (4.85,5.43)	5.05 (4.79,5.31)	-0.09 (-0.49,0.3)	0.64	0.16 (-0.14,0.46)	0.29
	-	CS/SE	4.83 (4.57,5.09)	4.91 (4.68,5.14)	0.08 (-0.27,0.43)	0.65		
Hypercalcemia (%)	> 11 mg/dL	SS/UC	10.3 (4.41,16.1)	17.5 (12.3,22.7)	7.23 (-0.64,15.1)	0.07	7.91 (2.53,13.3)	0.004
		CS/SE	6.99 (3.03,10.9)	7.48 (4.02,10.9)	0.49 (-4.76,5.75)	0.85		
Hyperphosphatemia (%)	> 5 mg/dL	SS/UC	46.4 (38.7,54.1)	45.3 (38.5,52.2)	-1.08 (-11.4,9.25)	0.84	3.55 (-4.27,11.4)	0.37
		CS/SE	38.9 (31.7,46)	42.3 (36.1,48.5)	3.42 (-6.06,12.9)	0.48		
Subgroup of patients who received any type of MBD medication (N = 204)								
Calcium (mg/dL)	-	SS/UC	10 (9.63,10.4)	10.4 (10.1,10.7)	0.38 (-0.12,0.89)	0.13	0.46 (0.21,0.7)	<0.001
	-	CS/SE	9.7 (9.5,9.9)	9.7 (9.53,9.86)	0.002 (-0.26,0.26)	0.99		
Phosphate (mg/dL)	-	SS/UC	5.39 (5.04,5.75)	5.24 (4.95,5.53)	-0.15 (-0.61,0.31)	0.52	0.36 (0.07,0.64)	0.01
	-	CS/SE	4.99 (4.71,5.27)	4.91 (4.67,5.14)	-0.09 (-0.45,0.28)	0.63		
Hypercalcemia (%)	> 11 mg/dL	SS/UC	14.6 (7.46,21.7)	19 (13.3,24.8)	4.47 (-4.69,13.6)	0.34	9.76 (3.79,15.7)	0.001
		CS/SE	7.58 (3.14,12)	7.84 (4.14,11.5)	0.25 (-5.53,6.04)	0.93		
Hyperphosphatemia (%)	> 5 mg/dL	SS/UC	32.3 (24.6,40.1)	42.9 (35.8,50)	10.6 (0.1,21.1)	0.048	-0.51 (-8.3,7.28)	0.9
		CS/SE	37.2 (30.1,44.4)	40.1 (33.7,46.6)	2.9 (-6.72,12.5)	0.55		
Subgroup of patients who received NCBPBs or calcimimetics (N = 182)								
Calcium (mg/dL)	-	SS/UC	9.94 (9.71,10.2)	10.2 (10,10.4)	0.27 (-0.03,0.56)	0.08	0.38 (0.15–0.61)	0.001
	-	CS/SE	9.73 (9.51,9.94)	9.69 (9.52,9.86)	-0.04 (-0.31,0.24)	0.78		
Phosphate (mg/dL)	-	SS/UC	5.37 (5,5.75)	5.24 (4.94,5.54)	-0.14 (-0.62,0.34)	0.58	0.16 (-0.19,0.5)	0.37
	-	CS/SE	5.08 (4.78,5.39)	5.08 (4.83,5.32)	-0.01 (-0.4,0.38)	0.97		
Hypercalcemia (%)	> 11 mg/dL	SS/UC	12 (7.06,16.9)	17.8 (12.8,22.7)	5.77 (-1.22,12.8)	0.11	6.28 (1.58,11)	0.009
		CS/SE	9.19 (5.94,12.4)	8.03 (4.78,11.3)	-1.15 (-5.75,3.44)	0.62		
Hyperphosphatemia (%)	> 5 mg/dL	SS/UC	32.2 (25.1,39.4)	44.7 (37.5,51.8)	12.44 (2.33,22.5)	0.02	2.05 (-5.78,9.88)	0.61
		CS/SE	30.2 (23.6,36.9)	42.5 (35.8,49.2)	12.24 (2.8,21.7)	0.01		

Data are presented as mean or mean difference (95% confidence interval); SS/UC, Social Security/Universal Coverage; CS/SE, Civil Servant/State Enterprise; MBD, mineral and bone disorder; NCBPBs, non-calcium-based phosphate binder.

**Fig 5:**
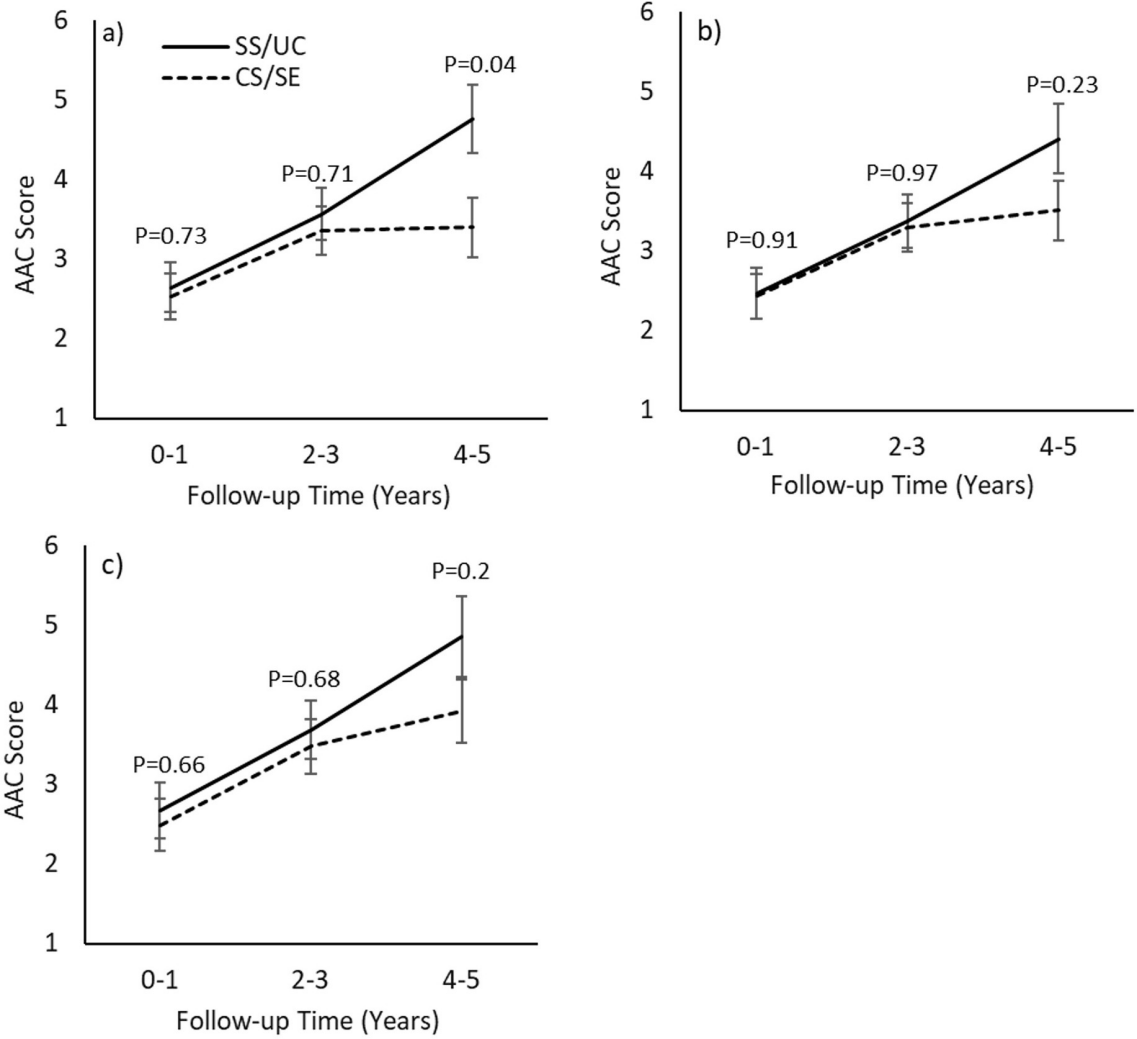
Changes in aortic arch calcification score of the propensity-score-matched subgroups. a) all patients; b) subgroup of patients who received any MBD medication; c) subgroup of patients who received NCBPBs or calcimimetics. P-values in the graphs represent the significance of the difference between two groups at each time point. AAC, aortic arch calcification; CS/SE, Civil Servant/State Enterprise; SS/UC, Social Security/Universal Coverage; MBD, mineral and bone disorder; NCBPBs, non-calcium-based phosphate binders.

Regarding the changes in AAC scores, an increase in the AAC score over time was evident in both groups across all propensity-score-matched subgroups (Table 6). The increase in the AAC score was more significant in magnitude in the SS/UC group, resulting in a substantially higher AAC score during the 4^th^-5^th^ year of follow-up in the SS/UC group compared with the CS/SE group in the matched subgroup derived from all patients (Fig 5). However, between-group changes in AAC scores were not significant in any of the propensity-score-matched subgroups.

**Table 6:** Changes in aortic arch calcification score.

Time from enrollment (Years)	0–1	2–3	4–5	Between-group Comparisons (SS/UC vs. CS/SE)	*P*
All patients					
SS/UC	2.65 (2.05,3.25)	3.57^a^ (2.92,4.21)	4.76^c^ (3.91,5.61)	0.2 (-0.62,1.02)	0.63
CS/SE	2.53 (1.97,3.1)	3.37^a^ (2.77,3.97)	3.4 (2.65,4.15)		
Subgroup of patients who received any type of MBD medication					
SS/UC	2.47 (1.84,3.09)	3.38 (2.7,4.05)	4.41^c^ (3.54,5.27)	0.06 (-0.76,0.88)	0.89
CS/SE	2.43 (1.86,3)	3.29^a^ (2.7,3.89)	3.51^a^ (2.76,4.26)		
Subgroup of patients who received NCBPBs or calcimimetics					
SS/UC	2.68 (1.98,3.37)	3.69 (2.95,4.42)	4.86^c^ (3.87,5.84)	0.16 (-0.76,1.08)	0.74
CS/SE	2.49 (1.85,3.13)	3.48^a^ (2.81,4.14)	3.92^b^ (3.13,4.71)		

Data are presented as mean or mean difference (95% confidence interval); SS/UC, Social Security/Universal Coverage; CS/SE, Civil Servant/State Enterprise; MBD, mineral and bone disorder; NCBPBs, non-calcium-based phosphate binders; ^a^p<0.05; ^b^P<0.01 and ^c^P<0.001 vs. Year 0–1 of the same group.

## Discussion

This is the first real-world study to examine the differences in mineral outcomes between two groups of patients receiving different coverage for MBD medication. The SS/UC program covered only CBPBs and active vitamin D, whereas the CS/SE program covered CBPBs, active vitamin D, NCBPBs, and calcimimetics. The important findings included serum calcium levels and the proportions of patients with hypercalcemia, which were substantially and invariably higher in the SS/UC group compared with the CS/SE group. Serum phosphate and the proportions of patients with hyperphosphatemia were mostly higher in the SS/UC group. The SS/UC group showed a more severe degree of hyperparathyroidism, and more patients in the SS/UC group reached the composite endpoint of parathyroidectomy and severe hyperparathyroidism. The progression of AAC was observed in both groups. A more rapid progression was suggested in the SS/UC group, but between-group changes were not statistically significant.

Previous studies have demonstrated a clear association between the use of CBPBs with increasing serum calcium and hypercalcemia in dialysis patients [20]. This was largely the result of the kidney’s inability to excrete excess calcium, especially in those taking high doses of CBPBs [21]. Moreover, the use of active vitamin D to control hyperparathyroidism also led to an increase in the absorption of calcium through the digestive tract, resulting in a further elevation of serum calcium levels [22,23]. The data from the present study confirmed these findings. The present study also demonstrated a progressive increase in serum calcium levels and in the proportion of patients with hypercalcemia with increasing dialysis vintage in the SS/UC group but not in the CS/SE group. This could be the result of a more rapid progression of hyperparathyroidism in the SS/UC group. Higher PTH levels cause more bone resorption, resulting in a release of more calcium into circulation.

The adverse health impacts of calcium load in dialysis patients have been emphasized. The use of CBPBs and higher serum calcium are associated with an increased risk of death [24,25]. The prolonged exposure to a high calcium environment causes the accumulation of excess calcium in the soft tissue and blood vessels, resulting in soft tissue and vascular calcification [5,7]. High prevalence and severe degree of coronary artery calcification are well-documented in dialysis patients [26,27]. The presence of arterial calcification leads to an increase in arterial stiffness, cardiovascular events, and mortality [28]. Due to this overwhelming evidence, avoiding hypercalcemia is recommended in dialysis patients [29].

As for phosphate control, serum phosphate levels and the proportions of patients with hyperphosphatemia were significantly higher in the SS/UC group in most models. These findings suggest inferior phosphate control in the SS/UC group compared with the CS/SE group. The percentages of patients with hyperphosphatemia were mostly stable throughout the study period in both groups except in the propensity-score matched subgroup of NCBPBs or calcimimetics. The increase in the proportions of patients with hyperphosphatemia with increasing dialysis vintage in this propensity-score matched subgroup was likely due to the fact these patients had a more severe degree of MBD, to begin with; therefore, they required the use of NCBPBs or calcimimetics for better control of MBD.

Although clinical studies have demonstrated equivalent or better efficacy of CBPBs compared with NCBPBs, it is possible that high serum calcium prevented an increase in the dose of CBPBs, resulting in less optimum phosphate control in the group of patients who did not have access to NCBPBs [30,31]. The association between hyperphosphatemia and increased mortality in dialysis patients has been well-documented [32,33]. This has been largely attributed to the phosphate-driven increase in vascular calcification and accumulation of fibroblast growth factor-23 [34,35]. Despite the current recommendation to lower serum phosphate levels towards the normal range, high-quality evidence based on randomized-controlled trials is still lacking [29]. Two large pragmatic trials to determine the optimum serum phosphate targets are currently ongoing [36].

Progression of hyperparathyroidism with increasing dialysis vintage occurred in both groups of patients. The SS/UC group had substantially higher PTH levels compared with the CS/SE group in most models. More patients in the SS/UC group reached the composite outcome of parathyroidectomy and severe hyperparathyroidism. After cinacalcet became available, a decline in the rate of parathyroidectomy has been observed in several countries and regions [37]. As an allosteric activator of the calcium-sensing receptor, cinacalcet inhibits PTH release, as well as lowers serum calcium, making the drug ideal for patients with hyperparathyroidism and hypercalcemia [38]. Although only 36% of the patients received calcimimetics in the present study, a total of 70% received NCBPBs and/or calcimimetics. The reduction in serum calcium by NCBPBs most likely allowed the dose of active vitamin D to be titrated up, providing better control of hyperparathyroidism. Together, NCBPBs and calcimimetics helped in controlling PTH levels in the CS/SE group.

Since NCBPBs and calcimimetics may attenuate the progression of vascular calcification, changes in AAC were analyzed in three propensity-score-matched subgroups. More rapid progression of AAC was suggested in the SS/UC group, but between-group changes were not significant. The lack of significant differences in the progression of AAC between the two groups could be partly due to a small number of patients as well as the semiquantitative and subjective methods used in the assessment. Additionally, the combined use of CBPBs and/or active vitamin D with NCBPBs and/or calcimimetics in the same patient, either simultaneously or at different time intervals, could also influence the development and progression of vascular calcification in the long term.

The present study is limited by substantial differences in baseline characteristics between the two groups of patients under different healthcare programs. To overcome this limitation, adjusted analyses and propensity-score matching before analyses were applied. However, this method might not be able to eliminate all potential confounders. Sixty-three percent of the patients in the SS/UC group were under the UC program. Some patients under the UC program might be less educated or have lower socioeconomic status resulting in a higher rate of non-compliance to dietary restrictions and medications. Additionally, while the analysis of AAC was performed using a chest X-ray, more objective and quantitative results of vascular calcification could be obtained from computed tomography.

In conclusion, the group of patients under the healthcare program that did not cover the use of NCBPBs and calcimimetics showed worse mineral outcomes, including higher serum calcium and phosphate levels and a more rapid progression of hyperparathyroidism compared to the group of patients under the healthcare program that covered the use of NCBPBs and calcimimetics. The data from the present study could not confirm the difference in the progression of vascular calcification between the two groups.

## Supporting information

S1 Table Pearson correlations between age and laboratory data. (PDF)

S2 Table Relationships between age and demographic data. (PDF)

S3 Table Relationships between sex with demographic and laboratory data. (PDF)

S4 Table Relationships between diabetes with demographic and laboratory data. (PDF)

S5 Table Baseline characteristics of the subgroups of patients after propensity-score matching. (PDF)

S1 Fig Proportions of patients with abnormal mineral parameters for the subgroup of patients who received MBD medication. a) serum calcium >10.5 mg/dL; b) serum calcium >11 mg/dL; c) serum phosphate >4.5 mg/dL; d) serum phosphate >5 mg/dL; e) parathyroid hormone levels >600 pg/mL; f) parathyroid hormone levels >1000 pg/mL. The number of available laboratory data is shown below each graph. SS/UC, Social Security/Universal Coverage; CS/SE, Civil Servant/State Enterprise. *P<0.01 and **P<0.001 vs. Year 0–2 of the same group. P-values in the graph represent the significance of between-group changes after adjustment for age, sex, and diabetes (Model 2).
(PDF)

S1 Dataset Minimal data set for Tables 2–6 and Figs 2–5. (ZIP)
