# Supplementary material for: The impact of accessibility to non-calcium-based phosphate binders and calcimimetics on mineral outcomes in patients receiving maintenance hemodialysis: A 10-year retrospective analysis of real-world data
Source: PLoS One. 2024 May 31;19(5):e0304649. doi: 10.1371/journal.pone.0304649 (PMC11142503; doi:10.1371/journal.pone.0304649)
Supplement: S1 Table — (PDF) [file pone.0304649.s001.pdf]

S1 Table Pearson correlations between age and laboratory data

		Age	BMI	PTH	Hb	Alb	CCa	PO4	Cr
Age (year)	*R*	1	0.058	-0.330	0.087	-0.525	-0.068	-0.484	-0.578
	P-value		0.127	**<0.001**	**0.02**	**<0.001**	0.069	**<0.001**	**<0.001**
Body mass index (g/m^2)	*R*	0.058	1	-0.001	-0.011	-0.112	-0.036	0.108	0.051
	P-value	0.127		0.970	0.765	0.003	0.335	0.004	0.174
Parathyroid hormone (pg/mL)	*R*	-0.330	-0.001	1	-0.110	0.096	0.148	0.341	0.184
	P-value	<0.001	0.970		0.004	0.010	<0.001	<0.001	<0.001
Hemoglobin	*R*	0.087*	-0.011	-0.110	1	0.167	0.018	0.000	-0.045
	P-value	0.02	0.765	0.004		<0.001	0.637	0.992	0.228
Albumin (g/L)	*R*	-0.525	-0.112	0.096	0.167	1	-0.041	0.262	0.393**
	P-value	<0.001	0.003	0.010	<0.001		0.272	<0.001	<0.001
Corrected calcium (mg/dL)	*R*	-0.068	-0.036	0.148	0.018	-0.041	1	0.077	-0.027
	P-value	0.069	0.335	<0.001	0.637	0.272		0.039	0.479
Phosphate (mg/dL)	*R*	-0.484	0.108	0.341	<0.001	0.262	0.077	1	0.539
	P-value	<0.001	0.004	<0.001	0.992	<0.001	0.039		<0.001
Creatinine (mg/dL)	*R*	-0.578	0.051	0.184	-0.045	0.393	-0.027	0.539	1
	P-value	<0.001	0.174	<0.001	0.228	<0.001	0.479	<0.001	

Laboratory data were 12-month average values; Age was the age at the time of enrollment.
