# Supplementary material for: The impact of accessibility to non-calcium-based phosphate binders and calcimimetics on mineral outcomes in patients receiving maintenance hemodialysis: A 10-year retrospective analysis of real-world data
Source: PLoS One. 2024 May 31;19(5):e0304649. doi: 10.1371/journal.pone.0304649 (PMC11142503; doi:10.1371/journal.pone.0304649)
Supplement: S2 Table — (PDF) [file pone.0304649.s002.pdf]

S2 Table Relationships between age and demographic data

		N	Mean Age	Standard Deviation	P-value
Diabetes	No	457	51.2274	18.21291	<0.001
	Yes	257	65.2085	13.40295	
Cardiovascular disease	No	518	52.0774	17.55961	<0.001
	Yes	196	67.3135	13.77099	
Dyslipidemia	No	461	51.6429	17.63045	<0.001
	Yes	253	64.6725	15.26038	

Age was the age at the time of enrollment.
