# Supplementary material for: The impact of accessibility to non-calcium-based phosphate binders and calcimimetics on mineral outcomes in patients receiving maintenance hemodialysis: A 10-year retrospective analysis of real-world data
Source: PLoS One. 2024 May 31;19(5):e0304649. doi: 10.1371/journal.pone.0304649 (PMC11142503; doi:10.1371/journal.pone.0304649)
Supplement: S3 Table — (PDF) [file pone.0304649.s003.pdf]

S3 Table Relationships between sex with demographic and laboratory data

Labs	Sex	N	Mean	Standard Deviation	P-value
Body mass index (g/m^2)	Male	347	24.0325	4.86040	0.757
	Female	367	23.9016	6.24512	
Parathyroid hormone (pg/mL)	Male	347	543.7186	675.87291	0.322
	Female	367	498.5108	534.98719	
Hemoglobin (g/dL)	Male	347	10.9119	1.59290	0.106
	Female	367	10.7335	1.34986	
Albumin (g/L)	Male	347	36.4561	4.08243	<0.001
	Female	367	34.7670	4.06280	
Corrected calcium (mg/dL)	Male	347	9.7258	0.68385	0.001
	Female	367	9.8940	0.71030	
Phosphate (mg/dL)	Male	347	5.0091	1.45842	0.005
	Female	367	4.7089	1.39745	
Creatinine (mg/dL)	Male	346	10.0837	3.50777	<0.001
	Female	367	7.9769	2.73713	

Laboratory data were 12-month average values
