# Supplementary material for: The impact of accessibility to non-calcium-based phosphate binders and calcimimetics on mineral outcomes in patients receiving maintenance hemodialysis: A 10-year retrospective analysis of real-world data
Source: PLoS One. 2024 May 31;19(5):e0304649. doi: 10.1371/journal.pone.0304649 (PMC11142503; doi:10.1371/journal.pone.0304649)
Supplement: S4 Table — (PDF) [file pone.0304649.s004.pdf]

S4 Table Relationships between diabetes with demographic and laboratory data

Labs	DM	N	Mean or Count	SD or %	P-Value
Age (year)	No	457	51.2274	18.21291	<0.001
	Yes	257	65.2085	13.40295	
Body mass index (g/m^2)	No	457	23.2013	5.40844	<0.001
	Yes	257	25.3225	5.70324	
Cardiovascular disease (n/%)	No	457	81	17.7	<0.001
	Yes	257	115	44.7	
Dyslipidemia (n/%)	No	457	112	24.5	<0.001
	Yes	257	141	54.9	
AV fistula (n/%)	No	457	285	62.4	<0.001
	Yes	257	99	38.5	
AV graft (n/%)	No	457	95	20.8	
	Yes	257	88	34.2	
Catheter (n/%)	No	457	77	16.8	
	Yes	257	70	27.2	
PTH (pg/mL)	No	457	618.9791	668.34795	<0.001
	Yes	257	347.1482	431.49147	
Hemoglobin (g/dL)	No	457	10.6905	1.53781	0.002
	Yes	257	11.0282	1.27599	
Albumin (g/L)	No	457	36.2381	4.28402	<0.001
	Yes	257	34.4317	3.65029	
Corrected calcium (mg/dL)	No	457	9.8893	0.71661	<0.001
	Yes	257	9.6753	0.65494	
Phosphate (mg/dL)	No	457	5.0374	1.47803	<0.001
	Yes	257	4.5301	1.29338	
Creatinine (mg/dL)	No	457	9.6901	3.33044	<0.001
	Yes	257	7.7661	2.87806	

Laboratory data were 12-month average values; Age was the age at the time of enrollment
