# Supplementary material for: The impact of accessibility to non-calcium-based phosphate binders and calcimimetics on mineral outcomes in patients receiving maintenance hemodialysis: A 10-year retrospective analysis of real-world data
Source: PLoS One. 2024 May 31;19(5):e0304649. doi: 10.1371/journal.pone.0304649 (PMC11142503; doi:10.1371/journal.pone.0304649)
Supplement: S5 Table — (PDF) [file pone.0304649.s005.pdf]

S5 Table Baseline characteristics of the subgroups of patients after propensity-score matching

Parameters	All patients			Subgroup of patients who received any MBD medication			Subgroup of patients who received NCBPBs or calcimimetics		
	SS/UC N=116	CS/SE N=116	*P*	SS/UC N=102	CS/SE N=102	*P*	SS/UC N=91	CS/SE N=91	*P*
Age (year)	53.99±12.24	54.05±11.85	0.97	52.22±12.65	53.36±12.3	0.51	52.8±13.2	54±11.5	0.49
Male sex (n/%)	63 (54.3)	71 (61.2)	0.29	56 (54.9)	59 (57.8)	0.67	49 (53.8)	53 (58.2)	0.55
Body mass index (kg/m^2)	23.44±5.3	24.24±4.13	0.2	23.59±4.9	24.68±4.58	0.11	23.68±4.08	24.62±3.99	0.12
Diabetes mellitus (n/%)	39 (33.6)	40 (34.5)	0.89	29 (29.4)	34 (33.3)	0.45	30 (33)	29 (31.9)	0.87
Cardiovascular disease (n/%)	33 (28.4)	24 (20.7)	0.17	25 (24.5)	19 (18.6)	0.31	24 (26.4)	18 (19.8)	0.29
Hypertension (n/%)	104 (89.7)	99 (85.3)	0.32	90 (88.2)	89 (87.3)	0.83	81 (89)	79 (86.8)	0.65
Dyslipidemia (n/%)	40 (34.5)	42 (36.2)	0.78	39 (38.2)	34 (33.3)	0.47	34 (37.4)	29 (31.9)	0.44
Dialysis vintage (months)	20.8 (9.13-57.1)	22.5 (7.02-56.4)	0.99	26.4 (11.1-72.3)	24.8 (6.72-61.3)	0.3	24 (12.1-66.9)	23.4 (9.1-67.6)	0.71
Arteriovenous Access (n/%)			0.02			0.04			0.03
Arteriovenous fistula	74 (63.8)	57 (49.1)		70 (68.6)	53 (52)		65 (71.4)	49 (53.8)	
Arteriovenous graft	21 (18.1)	39 (33.6)		19 (18.6)	33 (32.4)		14 (15.4)	28 (30.8)	
Catheter	21 (18.1)	20 (17.2)		13 (12.7)	16 (15.7)		12 (13.2)	14 (15.4)	
CKD-MBD Medication (n/%)									
CBPBs	76 (65.5)	73 (62.9)	0.68	83 (81.4)	69 (67.6)	0.03	75 (82.4)	56 (61.5)	0.002
NCBPBs	6 (5.2)	63 (54.3)	<0.001	6 (5.9)	64 (62.7)	<0.001	0 (0)	76 (83.5)	<0.001
Active vitamin D	69 (59.5)	59 (50.9)	0.19	67 (65.7)	50 (49)	0.02	62 (68.1)	48 (52.7)	0.03
Calcimimetics	4 (3.4)	44 (37.9)	<0.001	3 (2.9)	45 (44.1)	<0.001	0 (0)	49 (53.8)	<0.001
NCBPBs or calcimimetics	7 (6)	75 (64.7)	<0.001	6 (5.9)	74 (72.5)	<0.001	0 (0)	91 (100)	<0.001
Any CKD-MBD medication	98 (84.5)	105 (90.5)	0.17	102 (100)	102 (100)	-	91 (100)	91 (100)	-
Laboratory Data									
Hemoglobin (g/dL)	10.91±1.59	10.99±1.54	0.71	10.89±1.64	10.88±1.56	0.95	10.8±1.62	11.1±1.33	0.2
Albumin (g/L)	36.32±3.31	35.93±3.37	0.37	36.52±3.3	36.14±3.72	0.44	36.6±3.46	36.1±3.21	0.32
Calcium (mg/dL)	9.95±0.8	9.75±0.75	0.054	9.95±0.8	9.74±0.76	0.058	9.89±0.77	9.74±0.76	0.19
Phosphate (mg/dL)	5.14±1.4	4.99±1.36	0.41	5.28±1.51	5.08±1.34	0.32	5.31±1.51	5.23±1.28	0.7
PTH (pg/mL)	350 (163-713)	362 (181-591)	0.91	420 (221-748)	375 (181-663)	0.39	364 (198-761)	407 (211-695)	0.99
Creatinine (mg/dL)	9.98±2.98	9.48±2.87	0.19	10.24±2.93	9.78±2.69	0.24	10.4±3.04	9.95±2.61	0.26
Aortic Arch Calcification									
Score >0 (n/%)	69 (59.5)	69 (59.5)	0.99	63 (61.8)	64 (62.7)	0.93	58 (63.7)	57 (64)	0.97
Score	2.44±3.05	2.33±2.86	0.9	2.26±2.9	2.27±2.55	0.74	2.47±3.03	2.3±2.65	0.93

Laboratory data were 12-month average values. SS/UC, Social Security/Universal Coverage; CS/SE, Civil servant/State enterprise; MBD, mineral and bone disorder; CBPBs, calcium-based phosphate binders; NCBPBs, non-calcium-based phosphate binders; PTH, parathyroid hormone
