# Supplementary material for: The impact of accessibility to non-calcium-based phosphate binders and calcimimetics on mineral outcomes in patients receiving maintenance hemodialysis: A 10-year retrospective analysis of real-world data
Source: PLoS One. 2024 May 31;19(5):e0304649. doi: 10.1371/journal.pone.0304649 (PMC11142503; doi:10.1371/journal.pone.0304649)
Supplement: S1 Fig — a) serum calcium >10.5 mg/dL; b) serum calcium >11 mg/dL; c) serum phosphate >4.5 mg/dL; d) serum phosphate >5 mg/dL; e) parathyroid hormone levels >600 pg/mL; f) parathyroid hormone levels >1000 pg/mL. The number of available laboratory data is shown below each graph. SS/UC, Social Security/Universal Coverage; CS/SE, Civil Servant/State Enterprise. *P<0.01 and **P<0.001 vs. Year 0–2 of the same group. P-values in the graph represent the significance of between-group changes after adjustment for age, sex, and diabetes (Model 2). (PDF) [file pone.0304649.s006.pdf]

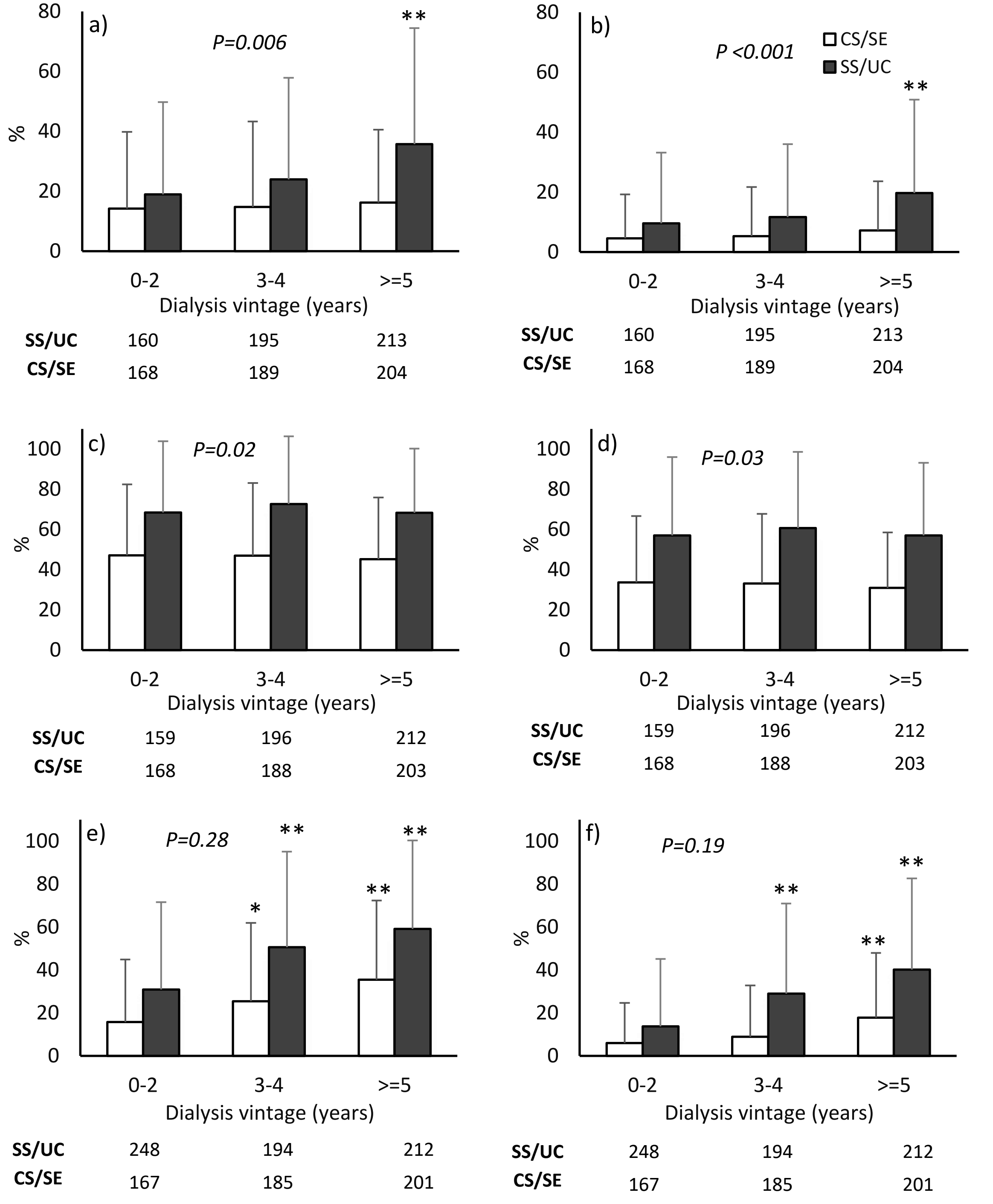

S1 Figure Proportions of patients with abnormal mineral parameters for the subgroup of patients who received MBD medication

a) serum calcium >10.5 mg/dL; b) serum calcium >11 mg/dL; c) serum phosphate >4.5 mg/dL; d) serum phosphate >5 mg/dL; e) parathyroid hormone levels >600 pg/mL; f) parathyroid hormone levels >1000 pg/mL

The number of available laboratory data is shown below each graph. SS/UC, Social Security/Universal Coverage; CS/SE, Civil Servant/State Enterprise

**P<0.01* and ***P<0.001* vs. Year 0-2 of the same group. P-values in the graph represent the significance of between-group changes after adjustment for age, sex, and diabetes (Model 2)
